# 

**Published:** 1811-07

**Authors:** 


### MEDICAL AND PHYSICAL

### JOURNAL.

CONDUCTED BY

SAMUEL FOTHERGILL, M.D.

AND

WILLIAM ROYSTON, ESQ.

VOL. XXVI.

FROM JULY TO DECEMBER, 1811.

Et quoniam variant morbi, variabimus artes;

Mille mali species, mille salutis erunt.

*LONDON:*

PRINTED FOR RICHARD PHILLIPS, 47, LUDGATE-HILL;

BY J. ADLARD, 23, BARTHOLOMEW-CLOSE.

W
1
ME 182

[Entered at Stationers' Hall.]

### MONTHLY CATALOGUE OF MEDICAL BOOKS.

A Treatise on Surgical Anatomy ; by A. Colles, one of the Professors of Anatomy and Surgery in the Royal College of Surgeons in Ireland, &c. &c. &c. Part 1st. 8vo. Longman and Co.

A Letter respectfully addressed to the Commissioners for Transports, sick and wounded Seamen, &c. &c. &c. on the Subject of the Operation for Popliteal Aneurism ; Illustrated by Cases and a description of a new Instrument. By A. C. Hutchison, M. D. Surgeon to the Royal Naval Hospital at Deal 8vo. Callow.

A Posologic Companion to the London Pharmacopœia. By John Nott, M. D. and Member of the Royal College of Physicians in London. The Third Edition, adapted to the latest reform of the College, 18mo. Callow.

A Letter to Dr. Jones on the composition of the Eau Medicinale D'Husson. By James Moore, Member of the Royal College of Surgeons, 8vo. Johnson.

The Edinburgh New Dispensatory, including Translations of the London Pharmacopœia, published in 1809 ; the Edinburgh of 1805 ; and the Dublin of 1807. By A Duncan, Jun. M. D. Sixth Edition, corrected and enlarged. 8vo. Murray.

The New London Practice of Physic, pointing out the Characters, Causes, and Symptoms of the various Diseases to which the Human Body is liable. The Seventh Edition enlarged and carefully revised. By E. G. Clarke, M. D. 8vo. Cox.

### IN THE PRESS.

A Treatise on the Gout, containing the Opinions of the most celebrated Ancient and Modern Physicians on that Disease, with observations on the Eau Medicinale d'Husson. By John Ring, Member of the Royal College of Surgeons, London and Paris.

### NOTICES TO CORRESPONDENTS.

Mr. Harrup will perceive by this Number that the Editors have not been inattentive to the well-marked Case transmitted by him: but in the hurry with which the last pages of the Journal must necessarily be got up, the name of a gentleman (Harrold) to whom they are frequently indebted for valuable communications, was inserted instead of Harrup.

The Editors acknowledge the receival of some interesting statements from Dr. Hossack, respecting the Elgin Botanic Garden in the vicinity of New York, and of the *Hortus Elginensis*. From Dr. Spalding of Portsmouth, New Hampshire, they have also received some valuable meteorological details; they have much satisfaction in remarking the returning credit of the Medical and Physical Journal in the United States, and beg to assure their brethren of that country, that they will make every exertion to merit patronage, by collecting the most valuable materials that come within their reach, by liberal observations in their critical department, and by an honourable acknowledgment of the obligations they may be under to other periodical and printed works. Communications have been received from A. B. Granville, M. D. from H. R. on Dr. Harrison's Bill; from Mr. Whately, W. H., Mr. Smith, in continuation of the "Theory of Sensation," from A. B. G. from R. from A. Fogo, &c. &c.

We are much concerned that the observations of *Reprehensor* should be thought by Mr. Barlow unworthy of notice, because they are anonymous, and regret particularly their not having a real signature, if that circumstance prevents Mr. Barlow's further prosecution of an interesting inquiry. The public, also, are too well acquainted with the value of Mr. Barlow's opinions, and of his extensive practical information, not to feel with ourselves that his silence is a real loss. The support taken from the assertions of Dr. Douglass, in opposition to the opinions of Mr. Barlow, on the contagious property of the inoculated Small-pox, have, in themselves, but little value. Dr. Douglass was a violent prejudiced partizan, and his disingenuous and cruel opposition to Dr. Boylston, the intrepid introducer of Small-pox inoculation into America, will never be forgotten. Douglass was the Rowley of 1722: the rude and uncandid reprobater of a practice he did not himself approve. We apprehend that Reprehensor might have taken much stronger ground on the calculations of Dr. Rast of Vienna, and on the controversy which it produced, than on the suspected evidence of a mortal enemy to Inoculation, given at a period, when neither its merits nor its demerits were understood.

